# Supplementary material for: Cytokine-Induced iNOS in A549 Alveolar Epithelial Cells: A Potential Role in COVID-19 Lung Pathology
Source: Biomedicines. 2023 Oct 3;11(10):2699. doi: 10.3390/biomedicines11102699 (PMC10603955; doi:10.3390/biomedicines11102699)
Supplement: Supplementary file 1 [file biomedicines-11-02699-s001.zip › biomedicines-2609052-supplementary.pdf]

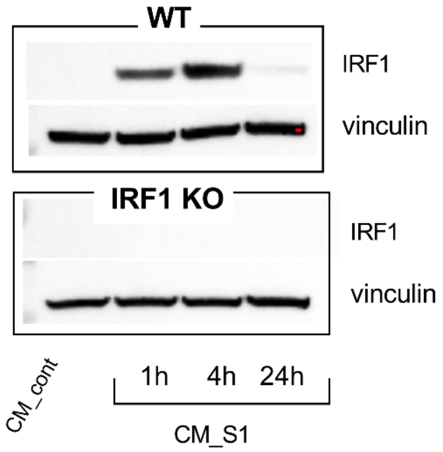

Figure S1. Validation of IRF1 KO cells. A549 WT and IRF1 KO were incubated in CM_cont or CM_S1. At the times indicated the expression of IRF1 protein was assessed by means of Western Blot analysis; representative blots are shown of three different experiments.

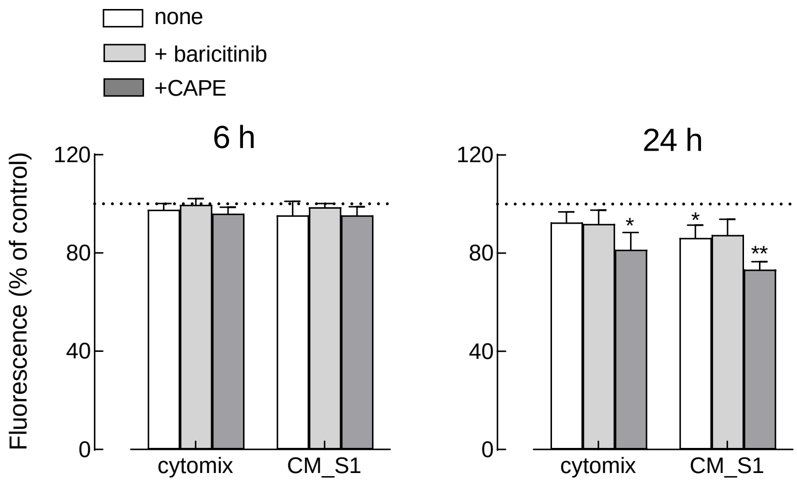

Figure S2. A549 WT cell viability. At the indicated times, cell viability was determined through the resazurin assay, as described in Methods, and expressed as percent of viability in cells incubated with CM_cont. Each bar represents the mean ± SEM of three independent experiments each performed in triplicate. *p<0.05; **p<0.01 vs CM_cont (=100, dotted line) with One sample t test.
